# Consensus on the Treatment and Follow-Up for Metastatic Castration-Resistant Prostate Cancer: A Report From the First Global Prostate Cancer Consensus Conference for Developing Countries (PCCCDC)

**DOI:** 10.1200/GO.20.00511

**Published:** 2021-04-15

**Authors:** Fernando Cotait Maluf, Felipe Moraes Toledo Pereira, Adriano Gonçalves Silva, Aldo Lourenço Abbade Dettino, Ana Paula Garcia Cardoso, André Seeke Sasse, Andrey Soares, Ariel Galapo Kann, Daniel Herchenhorn, Denis Leonardo Fontes Jardim, Diego Emilio Lopera Cortés, Fábio Roberto Kater, Igor A. Protzner Morbeck, João Francisco Navarro Reolon, José Augusto Rinck Jr, Juan Jose Zarbá, Juan Pablo Sade, Karine Martins da Trindade, Leonardo Atem G. A. Costa, Lucas V. dos Santos, Manuel Caitano Maia, Mariana Bruno Siqueira, Silke Gillessen

**Affiliations:** ^1^ Hospital Israelita Albert Einstein, São Paulo, Brazil; ^2^ Beneficência Portuguesa de São Paulo, São Paulo, Brazil; ^3^ Latin American Oncology Group (LACOG), Porto Alegre, Brazil; ^4^ Hospital São Camilo, São Paulo, Brazil; ^5^ Oncologia Clínica ICTr, Curitiba, Brazil; ^6^ Hospital AC Camargo, São Paulo, Brazil; ^7^ Grupo Sonhe, Campinas, Brazil; ^8^ Centro Paulista de Oncologia, Oncoclínicas, São Paulo, Brazil; ^9^ Hospital Alemão Oswaldo Cruz, São Paulo, Brazil; ^10^ Grupo de oncologia D'Or, Rio de Janeiro, Brazil; ^11^ Instituto D'Or de ensino e pesquisa, Rio de Janeiro, Brazil; ^12^ Hospital Sírio Libanês, São Paulo, Brazil; ^13^ Clínica Oncólogos del Occidente SAS, Manizales, Colombia; ^14^ Hospital Sírio Libanês, Brasília, Brazil; ^15^ Universidade Católica de Brasília, Brasília, Brazil; ^16^ Hospital Zenon Santillán, Nacional University of Tucumán, Tucumán, Argentina; ^17^ Instituto Alexander Fleming y de la Universidad Austral, Buenos Aires, Argentina; ^18^ IEP—Instituto de Ensino e Pesquisa Oncocentro, Fortaleza, Brazil; ^19^ Centro de Oncologia do Paraná, Curitiba, Brazil; ^20^ Oncology Institute of Southern Switzerland (IOSI), Bellinzona and Università della Svizzera Italiana, Lugano, Switzerland; ^21^ Manchester Cancer Research Centre, Division of Cancer Sciences, University of Manchester, Manchester, United Kingdom

## Abstract

**PURPOSE:**

To present a summary of the recommendations for the treatment and follow-up for metastatic castration-resistant prostate cancer (mCRPC) as acquired through a questionnaire administered to 99 physicians working in the field of prostate cancer in developing countries who attended the Prostate Cancer Consensus Conference for Developing Countries.

**METHODS:**

A total of 106 questions out of more than 300 questions addressed the use of imaging in staging mCRPC, treatment recommendations across availability and response to prior drug treatments, appropriate drug treatments, and follow-up, and those same scenarios when limited resources needed to be considered. Responses were compiled and the percentages were presented by clinicians to support each response. Most questions had five to seven relevant options for response including abstain and/or unqualified to answer, or in the case of yes or no questions, the option to abstain was offered.

**RESULTS:**

Most of the recommendations from this panel were in line with prior consensus, including the preference of a new antiandrogen for first-line therapy of mCRPC. Important aspects highlighted in the scenario of limited resources included the option of docetaxel as treatment preference as first-line treatment in several scenarios, docetaxel retreatment, consideration for reduced doses of abiraterone, and alternative schedules of an osteoclast-targeted therapy.

**CONCLUSION:**

There was wide-ranging consensus in the treatment for men with mCRPC in both optimal and limited resource settings.

## INTRODUCTION

Prostate cancer (PCa) is the second most common cancer in men,^1^ with 15% of men worldwide being diagnosed with it at some point during their life. Of those diagnosed, 80% will have a localized form and have a nearly 100% 5-year survival rate. The 20% patients remaining will have an advanced or metastatic form of the disease with only 26%-30% of them surviving 5 years.^2^ The initial management of PCa has for decades been based on androgen deprivation therapy (ADT) as its cancer cells are sensitive to the manipulation of testosterone and its metabolites.^3,4^ Reducing circulating testosterone to castrate levels (< 50 ng/dL) deprive the cells of their primary stimulus for growth^5^ and can induce PCa cell death.^6^ Unfortunately, PCa cells eventually become resistant to ADT because of different resistance mechanisms thereby becoming castration-resistant PCa. This form of the disease typically progresses rapidly, with patients dying within 2-4 years.^7,8^ Castration-resistant prostate cancer (CRPC) is defined by disease progression despite castrate levels of testosterone, and may present as either a continuous rise in serum prostate-specific antigen (PSA) levels, the progression of pre-existing disease, and/or the appearance of new metastases.^9^

**CONTEXT**

**Key Objective**
Generate a consensus on critical issues relevant to treatment of metastatic castration-resistant prostate cancer (PCa) focused in developing countries.
**Knowledge Generated**
Consensus was reached for metastatic castration-resistant prostate cancer of treatment-naive patients with docetaxel. For docetaxel-refractory cases, abiraterone reached consensus. Multiple other options were considered and reached consensus in cases of docetaxel-refractory disease in areas of limited resources including low-dose abiraterone, corticosteroids, ketoconazole, bicalutamide, mitoxantrone, and diethylstilbestrol.
**Relevance**
The voting results presented in this document can be used to support the treatment of metastatic castration-resistant PCa in areas of limited resources lacking specific guidelines.

In patients with CRPC who fail the initial therapy with curative intent (ie, radical prostatectomy, external beam radiotherapy, and brachytherapy), treatment options were once limited. Since 2004, chemotherapy is often used in patients with metastatic CRPC. Some landmark trials demonstrated an overall survival advantage of docetaxel compared with mitoxantrone, despite the marginal clinical benefit (2.4 months in TAX 327 and 1.9 months in SWOG 99-16; add *P* value).^10,11^ Currently, in the castration-resistant setting, since 2010, several key randomized controlled trials have demonstrated survival benefit with new therapies before and after docetaxel chemotherapy. Multiple new agents were approved by the US Food and Drug Administration (FDA) for the management of mCRPC, which all have varying mechanisms of action, namely sipuleucel-T,^12^ abiraterone acetate,^13,14^ enzalutamide,^15,16^ cabazitaxel,^17^ and radium-223.^18^ All these agents increased overall survival by some months, compared with the control group. New hormonal agents (abiraterone and enzalutamide) prolonged median survival by up to 3.9 and 4.8 months, respectively,^19^ chemotherapy treatment with docetaxel and cabazitaxel, often associated with significant side effects, prolonged overall survival by a few months,^11,15,20^ and treatment for diffused or painful bone metastases with radium-223 improved median overall survival by 3.6 months.^21^ Sipuleucel-T was only approved by the FDA and is not available elsewhere. Currently, FDA approved in 2020 olaparib and rucaparib, two poly ADP ribose polymerase inhibitors for adult patients with deleterious or suspected deleterious germline or somatic homologous recombination repair gene-mutated metastatic castration-resistant prostate cancer (mCRPC), which have progressed following prior treatment.^22,23^

With the incidence of PCa and burden of disease steadily increasing globally, healthcare systems, especially in regions of limited resources, will struggle with its management when balancing the cost-effectiveness of all innovative technologies for the overall healthcare system.^24^

The following manuscript will summarize the recommendations of a large panel of physicians from developing countries, specializing in PCa, regarding the treatment and follow-up of patients presenting with mCRPC both with and without contemplating the restrictions of limited resources in the decision-making process, with the objective of providing guidance in clinical practice and policy development and modification. The complete methodology of Prostate Cancer Consensus Conference for Developing Countries including the elaboration process of the questionnaires to guide the panelists, the design of voting sessions, and consensus criteria were presented in the editorial and are valid for all the manuscripts.

## STAGING

**FIG 1:**
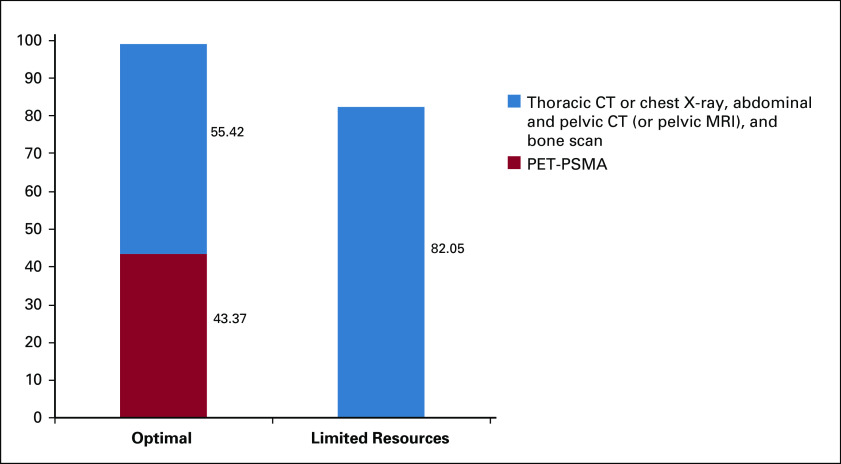
Recommendations for imaging for patients with metastatic castration-resistant prostate cancer (without and with limited resources). CT, computed tomography; PET-PSMA, positron emission tomography-prostate-specific membrane antigen.

The majority (55.42%) of experts recommended using a combination of thoracic computed tomography (CT) or chest X-ray, abdominal and pelvic CT (or pelvic MRI), and a bone scan to stage patients with mCRPC. Findings are summarized in Figure 1. As seen, in areas of limited resources, that number rises to more than 80% of experts recommending the use of multiple imaging, therefore reaching a consensus. By contrast, in best practices, 43% of experts recommended the use of positron emission tomography (PET)-CT with prostate-specific membrane antigen (PSMA) or PET-MRI. It is important to point out that PET-CT PSMA or PET-MRI is far more expensive than conventional imaging. Also, a recent study has not shown any treatment changes using next-generation image compared with conventional images in 35 patients with metastatic disease.^25^

## TREATMENT RECOMMENDATIONS

Table 1 summarizes physician responses for treatment recommendations and different scenarios when management options are limited because of resources. The best practice option is bolded, a dash (—) indicates that the treatment option was not offered as a response, a blank cell in the column had the treatment option offered though had zero physician respondents choosing that treatment, and the abbreviated NA-LR (not available because of limited resources) is an option eliminated to explore scenarios of limited resources.

**TABLE 1:**
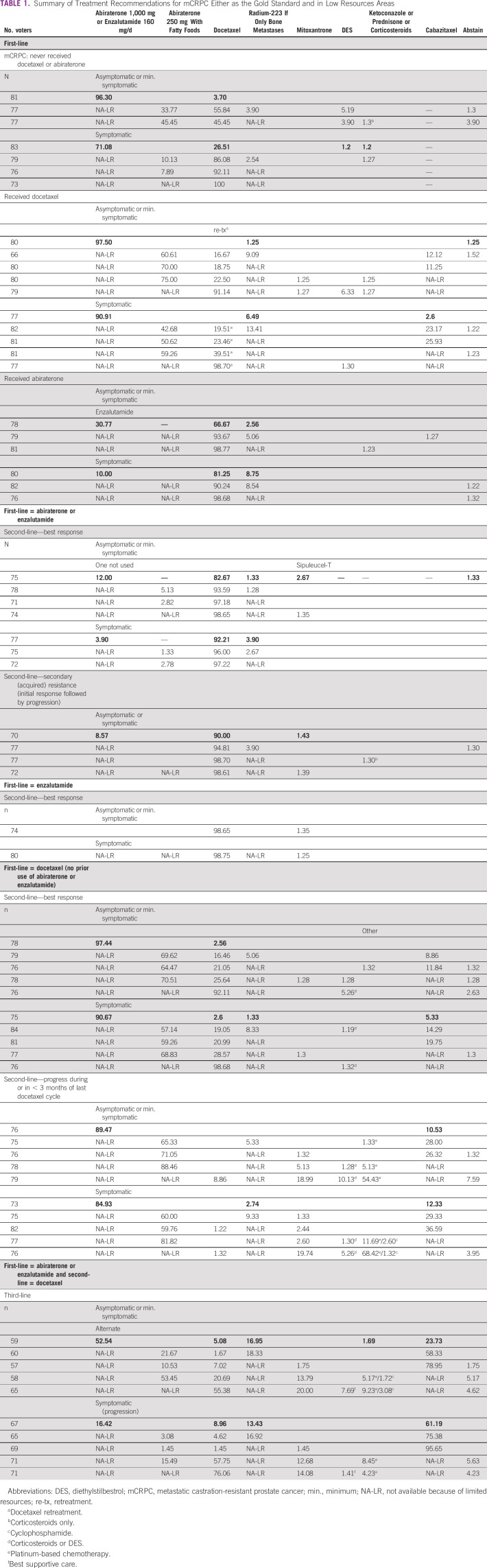
Summary of Treatment Recommendations for mCRPC Either as the Gold Standard and in Low Resources Areas

In cases when chemotherapy is not recommended, 86.6% of the panelists would recommend a first-generation AR antagonist—although not presented in Table 1, all questions are provided in the Data Supplement.

The preferred treatment option for patients who have never received docetaxel or abiraterone, or who had already received docetaxel, was abiraterone 1,000 mg/daily or enzalutamide 160 mg/daily. In cases when full doses were not available because of limited resources, reduced doses of abiraterone 250 mg/daily accompanied by a diet of fatty foods was a viable option for a substantial portion of physicians in all limited resource scenarios.

Many guidelines such as EAU-ESTRO-SIOG^20^ support the use of one of the following agents for the treatment of mCRPC (level 1 of evidence): abiraterone acetate plus prednisone, enzalutamide, radium-223, docetaxel at 75 mg/m^2^ every 3 weeks, and sipuleucel-T. By contrast, cabazitaxel, abiraterone acetate plus prednisone, enzalutamide, and radium are approved for second-line treatment of CRPC following docetaxel. A possible explanation for the lack of consensus in recommending abiraterone or enzalutamide as first systemic treatment for mCRPC for asymptomatic or minimally symptomatic men who did and did not receive docetaxel or abiraterone in the castration-sensitive or castration-naive setting may have to do with physicians' perception that both agents have a more favorable toxicity profile. By contrast, 55.8% of the panelists voted for docetaxel (no prior docetaxel exposure) in the same scenario if full doses of abiraterone and enzalutamide are not available. This can be explained by the fact that docetaxel is significantly less expensive than new hormonal therapy agents.^26,27^

The experts were asked to classify appropriate drugs for healthcare settings with limited resources that were on the WHO essential medicines list and/or could be sourced at an affordable price from a generic manufacturer. Overall, there was an agreement by the group, as Table 2 shows, with 10 of 12 drugs reaching a consensus, one with a near consensus, and the other with a 64% majority.

**TABLE 2:**
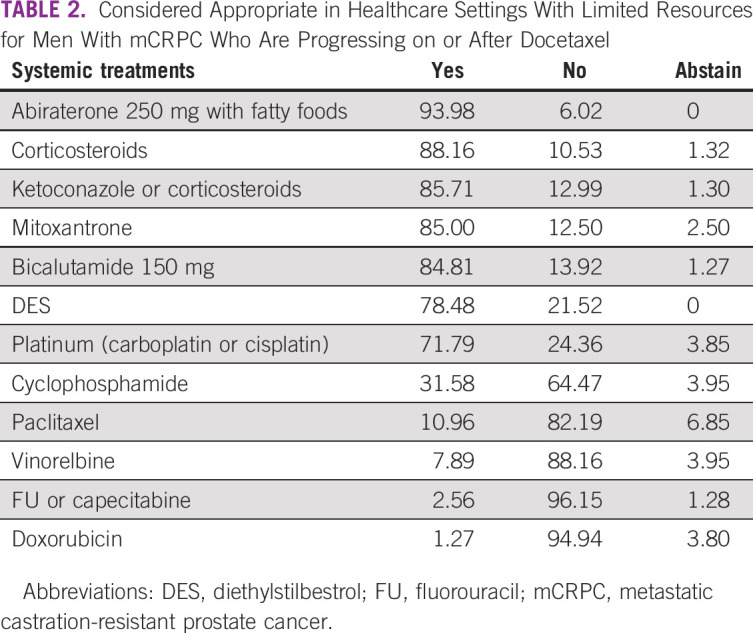
Considered Appropriate in Healthcare Settings With Limited Resources for Men With mCRPC Who Are Progressing on or After Docetaxel

A consensus was reached favoring low-dose abiraterone with fatty diet if full doses of abiraterone and enzalutamide as well as radium-223 and cabazitaxel were not available. The most robust data in this regard come from initial studies showing that in patients with mCRPC, abiraterone AUC was ∼2-fold higher with a high-fat meal and similar with a low-fat meal versus modified fasting state.^27^ These data were followed by a randomized phase II study including 72 patients with progressive CRPC, which compared low-dose abiraterone (250 mg qd) given with a low-fat meal versus standard-dose abiraterone (1,000 mg qd) under fasting conditions. Both arms received prednisone 5 mg twice daily. At 12 weeks, there was a greater effect on PSA in the low abiraterone arm (mean log change, −1.59) compared with standard dose (−1.19), and noninferiority of low abiraterone was established according to predefined criteria. The PSA response rate was 58% in low abiraterone and 50% in standard abiraterone arm, and the median progression-free survival was around 9 months in both groups.^28,29^

Bicalutamide or flutamide was preferred by 86.59% of the panelists as the second-line endocrine manipulation in conditions where neither abiraterone nor enzalutamide was available, and the patient is not a candidate for chemotherapy. This consensus may be explained by the popularity of these agents for many years associated with their low cost and side effects.^30^

In a different context, the panelists recommended as a consensus docetaxel retreatment for the majority of asymptomatic or minimally symptomatic men who did receive docetaxel in the castration-sensitive or castration-naive setting if abiraterone and enzalutamide as well as radium-223 and cabazitaxel are not available. Similarly, a consensus was reached in favor of docetaxel retreatment in either asymptomatic or minimally symptomatic or symptomatic patients who did receive abiraterone in the castration-sensitive or castration-naive setting in an area of limited resources if enzalutamide is unavailable. In a retrospective analysis from a trial that compared castration with or without docetaxel in the castration-sensitive setting showed that docetaxel retreatment was associated with limited activity with no predictive factors of response. In this retrospective analysis including 245 patients, the response rate to docetaxel retreatment in the CRPC scenario was only 20%. The median biochemical progression-free survival was only 4.1 months.^31^ Consequently, the panelists favored low-dose abiraterone (42.68%) over docetaxel retreatment (19.51%) in symptomatic patients when available.

For most symptomatic patients with mCRPC who did not receive docetaxel or abiraterone in the castration-sensitive or castration-naive setting, 71.08% of the panelists recommended abiraterone or enzalutamide. It is important to mention that both COU-AA-302^32^ and Prevail^17^ did not include truly symptomatic patients because the control arm was a placebo. However, there is no reason aside from trial design that would preclude the activity of both agents in this setting. By contrast, in limited resource areas where full doses of abiraterone and enzalutamide are not available, the panelists reached consensus in recommending docetaxel.^14^ When evaluating different drugs for the management of PCa that have no level 1 evidence regarding an overall survival benefit, the panelists reached a consensus favoring mitoxantrone or prednisone.^33^ Similarly, low-dose abiraterone with fatty diet was determined by consensus to be an adequate option to be used in this limited resource setting and is supported by the phase II randomized data.^29^

Other forms of hormonal therapy that reached consensus for their use in circumstances of limited resources were diethylstilbestrol, high-dose bicalutamide, ketoconazole, and corticosteroids alone. A phase II trial evaluated the activity of bicalutamide in doses ranging from 50 to 150 mg/d in 61 patients treated with combined androgen blockade. High-dose bicalutamide was associated with a PSA decline by 50% or more in 22% of patients. The median duration of response was 3.7 months and toxicity was mild.^34^ A recent review of the role of ketoconazole for the treatment of mCRPC showed that this agent is associated with low cost, a relatively favorable toxicity profile compared with chemotherapy, and its improved efficacy, both before and after chemotherapy, despite not showing a survival benefit.^35^ A recent review of the role of corticosteroids has indicated the relevance of this class of agents in their ability to manage adverse effects, reduce symptoms, and improve patients' quality of life.^36^ These options are selected based on their easy access and low costs, favorable toxicity profile, and will continue to be used as substitutes for newer agents not available in areas of limited resources.^37^

A total of 71% of the panelists recommended platinum compounds as appropriate treatment options in the setting of limited healthcare resources and in men with mCRPC who progressed on or after docetaxel. A systematic search on electronic databases evaluated the role of platinum compounds for mCRPC and suggested a statistically significant increase in both clinical as well biochemical response when adding platinum compounds to chemotherapy.^38^ Similarly, oral cyclophosphamide was favored by 64.47% of the panelists. A comprehensive literature search was performed and concluded that it is active in the treatment for CRPC even among patients previously treated with chemotherapy, including docetaxel, yielding symptomatic and objective responses.^39^

As second-line treatment, after prior docetaxel exposure, a near consensus (74.07%) was reached favoring the use of cabazitaxel at the dose of 20 mg/m^2^ intravenous every 3 weeks, with dose reductions in subsequent cycles as indicated in areas of limited resources. This recommendation is supported by a phase III study that assessed the noninferiority of cabazitaxel 20 mg/m^2^ versus 25 mg/m^2^. This trial showed that cabazitaxel 20 mg/m^2^ every 3 weeks was not inferior to higher dose in terms of overall survival. Health-related quality of life did not differ between cohorts.^40^

For second-line therapy, a consensus was reached favoring docetaxel in patients with metastatic asymptomatic or symptomatic CRPC who had progressive disease to first-line abiraterone or enzalutamide either when the resources were available or in areas with limited resources. Although there is no randomized trial comparing docetaxel versus abiraterone or enzalutamide in patients with mCRPC who failed prior enzalutamide or abiraterone, respectively, a recent review suggested a high degree of cross-resistance when abiraterone and enzalutamide are sequentially administered.^41^

Recently, the investigators of the COU-AA-302 trial described the activity of post-progression therapies in patients treated in the abiraterone experimental arm. The PFS of the patients who were treated with docetaxel as a first post-progression treatment was 7.6 months,^42^ whereas in those who received enzalutamide as a first post-progression treatment, it was 2.8 months.^43^ A retrospective report described the outcomes of 546 patients who progressed on first-line treatment with abiraterone or enzalutamide and subsequently received docetaxel or other hormone agents not previously administered as first-line. The authors reported that clinical and PSA response rates at both 3 and 6 months clearly favored docetaxel.^44^ Also, docetaxel is significantly less expensive than new hormonal therapy agents, which facilitates patient access.^26^

If low-dose abiraterone, cabazitaxel, and radium-223 were not available, there was a consensus favoring docetaxel retreatment in asymptomatic or minimally symptomatic men, who had response to docetaxel for mCRPC (without prior abiraterone or enzalutamide). It is important to mention that this option must be pursued if there are limitations related to access to other life-prolonging systemic agents as there are no randomized studies to support this strategy. A prospective phase II including 45 patients with mCRPC initially responding to docetaxel who then experienced disease progression after a period of biochemical remission of at least 5 months were enrolled and retreated with docetaxel. A total of 24.5% had a PSA response.^45^

Regarding third-line therapy, there was a consensus favoring cabazitaxel in symptomatic patients with mCRPC who had been treated with first-line abiraterone or enzalutamide and responded to docetaxel followed by progression if full doses of abiraterone and enzalutamide were not available or if full doses of abiraterone and enzalutamide as well as radium-223 were not available. A randomized phase III trial in patients with mCRPC who were previously treated with docetaxel and had progression within 12 months on abiraterone or enzalutamide compared cabazitaxel versus the alternative inhibitor (abiraterone or enzalutamide). The median overall survival was 13.6 months with cabazitaxel and 11.0 months with the androgen-signaling-targeted inhibitor (*P* = .008).^46^

By contrast, in limited resource situations, the preferred third-line mCRPC treatment option was docetaxel retreatment (consensus) in symptomatic patients with mCRPC who had been treated with first-line abiraterone or enzalutamide and responded to docetaxel, followed by progression if abiraterone and enzalutamide as well as radium-223 and cabazitaxel were not available. As seen in Table 3, most physicians recommend the use of cabazitaxel after abiraterone or enzalutamide and docetaxel treatments have been exhausted. There was a near consensus with 74% of physicians recommending a 20 versus 25 mg/m^2^ dose.

**TABLE 3:**
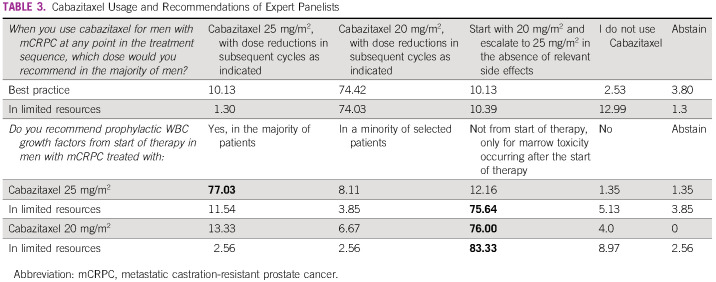
Cabazitaxel Usage and Recommendations of Expert Panelists

## OSTEOCLAST-TARGETED THERAPY

The last topic of the consensus referred to bone direct therapy to prevent bone-related complications. In areas of limited resources, a consensus was reached for the use of zoledronic acid over denosumab in patients with mCRPC and bone metastases for prevention of skeletal-related events (SRE) or symptomatic skeletal events (SSE), the cost of denosumab overcomes potential benefit.^47-51^ Table 4 summarizes the physicians' recommendations on this topic.

**TABLE 4:**
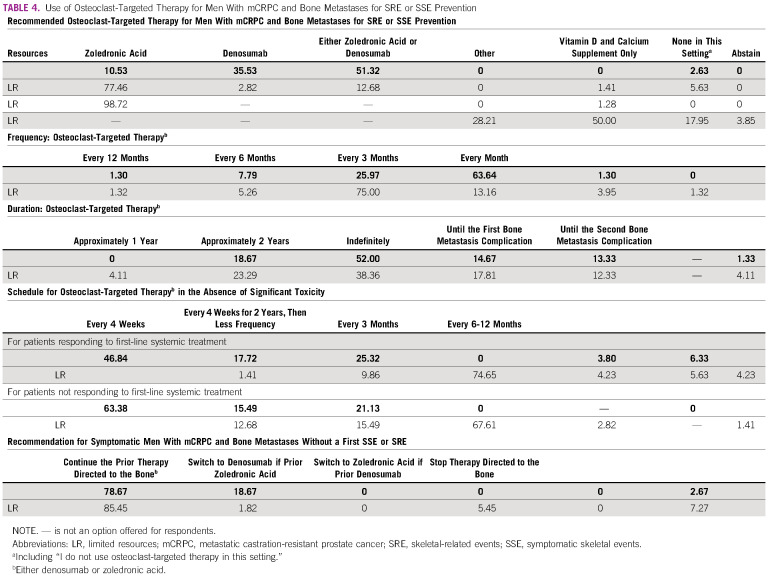
Use of Osteoclast-Targeted Therapy for Men With mCRPC and Bone Metastases for SRE or SSE Prevention

There was a consensus favoring the administration of zoledronic acid every 3 months instead of on monthly basis in areas of limited resources. This is supported by a randomized phase III trial including patients with metastatic breast cancer, metastatic PCa, or multiple myeloma who had at least one site of bone involvement. This study compared zoledronic acid administered intravenously every 4 weeks versus every 12 weeks for 2 years. The proportions of SREs did not differ significantly in both cohorts.^51^

## FOLLOW-UP

Regarding follow-up of patients with metastatic CRPC in areas of limited resources, most of the panelists (69.51%) would recommend only PSA every 1-2 months and image studies only in case of PSA elevation and/or symptoms suggesting clinical progression. This differs from the original recommendations as PSA is not a reliable surrogate marker in patients treated with some of the new agents such as abiraterone or enzalutamide.^52^ One explanation reflects the costs and access to frequent image tests.

In conclusion, the current study illustrates the consensus of physicians in the field of mCRPC treatment. With consensus levels of over 90% in many cases, including limited resource settings, the panelists made determinations that may elucidate treatment decisions and provide solutions for areas that face resource limitations. The use of docetaxel as first-line treatment for mCRPC or as retreatment is one of the most important results of this consensus, given that it entails a less expensive and popular agent. The utility of older agents in areas of resource limitations was not underestimated in the consensus and represents an important finding reflecting a trend toward accepting clinical benefit. Equally, prolonging the treatment interval of zoledronic acid was considered another reasonable solution when financial limitations were an issue. This consensus was developed with the aim of assisting developing countries in the treatment of mCRPC. This consensus concentrated a large group of experts located in South America. It is important to consider the socioeconomic differences of these countries in the incorporation of the results and consensus obtained.
